# Fabrication and characterization of tea polyphenol W/O microemulsion‐based bioactive edible film for sustained release in fish floss preservation

**DOI:** 10.1002/fsn3.2845

**Published:** 2022-04-21

**Authors:** Mengna Zhang, Qiaoling Zhao, Yanan Lin, Haifeng Wang, Ruofan Shui, Shitong Wang, Lijun Ge, Yunyan Li, Gongshuai Song, Jinyan Gong, Haixing Wang, Xi Chen, Qing Shen

**Affiliations:** ^1^ 12625 Collaborative Innovation Centre of Seafood Deep Processing Zhejiang Province Joint Key Laboratory of Aquatic Products Processing Institute of Seafood Zhejiang Gongshang University Hangzhou China; ^2^ College of Food Science and Technology Nanjing Agricultural University Nanjing China; ^3^ Zhejiang Provincial People’s Hospital Hangzhou China; ^4^ Zhoushan Institute of Food & Drug Control Zhoushan China; ^5^ 91616 Zhejiang Provincial Key Lab for Biological and Chemical Processing Technologies of Farm Product School of Biological and Chemical Engineering Zhejiang University of Science and Technology Hangzhou China; ^6^ Zhejiang Province Key Lab of Anesthesiology The Second Affiliated Hospital and Yuying Children’s Hospital of Wenzhou Medical University Wenzhou China

**Keywords:** controlled release, fish floss, nanoemulsion‐based edible coating, shelf life, water activity

## Abstract

A coated nanoemulsion (CNE)‐based edible film was fabricated on the surface of fish floss (FF) to extend its shelf life during storage. The antioxidant tea polyphenol (TPP) was embedded into W/O microemulsion, which was further encapsulated into multiple emulsion (Multi‐E) together with functional soluble dietary fiber (SDF). The physicochemical properties indicated that the nanoemulsion‐based edible film (NEF) improved the morphology of FF and reduced the crystallinity of the film by scanning electron microscopy (SEM) and X‐ray diffraction (XRD). The water vapor permeability increased gradually and rose to only 0.99% after 5 h, resulting in the water activity of FF at a low level (≤0.51) during the storage period. The TPP inside was released at a constant rate (≤18.10%) on the surface, and such a rate was accelerated in the simulated gastrointestinal environment, especially in intestine reaching 60.12% after 5 h of digestion. Besides, the effect of NEF on the flavor was also evaluated and the contents of ketones, phenols, and pyrazines increased, which displayed a regulating effect on the overall flavor of FF by blocking the external moisture and suppressing the microorganism activity. In summary, the NEF effectively enhanced the flavor and taste of FF, controlled the release of TPP, and reduced the water activity during the storage, thereby extending the shelf life.

## Graphical abstract

Coated nanoemulsion (CNE)‐based edible film was fabricated on fish floss (FF) for preservation. W/O microemulsion (W/O‐ME), multiple emulsion (Multi‐E), and nanoemulsion were prepared in sequence. Structure was characterized and physical and functional properties were evaluated.

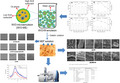

## 1 INTRODUCTION

Edible films or coatings are popularly applied in the preservation of foodstuffs, which are produced from a variety of natural and biodegradable materials (Tamimi et al., 2021). Edible films can act as protective barriers against weight loss and dampness, and modify internal gas composition to control microbiological growth, improve textural quality, and hide unpleasant odor (Paidari et al., 2021). Edible films are made of hydrocolloids and their blends have been extensively studied. For example, Alemán et al. (2016) developed an edible packaging cover by incorporating an active shrimp concentrate into a chitosan–gelatin matrix to preserve fish sausages during chilled storage. Albertos et al. (2019) used functional bio‐based seaweed edible films in the fish burgers to reduce microbial growth and control pH and water activity changes over storage. The edible films were demonstrated to be efficient in extending the freshness of foods (Jahdkaran et al., 2021). Therefore, researches on functional properties of edible films for specific application in aquatic products are still urgently needed.

Nanoemulsion is a colloidal system with kinetic stability composed of aqueous phase, oil phase, and surfactant (McClements, 2012). It is being increasingly applied in encapsulating, protecting, and delivering lipophilic ingredients to the beverage, fruits, meat, and related products. For example, essential oil was incorporated into nanoemulsion and immobilized with basil seed gum‐based film network to extend the shelf life of food products (Gahruie et al., 2017). Cinnamaldehyde nanoemulsion edible films based on pectin and papaya puree were elaborated for eco‐friendly and antimicrobial packaging material in food applications (Otoni et al., 2014). However, the application of this system in solid foods remains immature because of the challenge of the immobilization of nanoemulsions on the surface of foods (Salvia‐Trujillo et al., 2015). Therefore, nanoemulsion‐based edible films or coatings can represent an effective approach to dispose bioactive ingredients on the surface of various raw and/or processed foods and afford a controlled and efficient barrier, and thus achieve the purpose of improving quality and extending shelf life (Chang et al., 2021; Gahruie et al., 2017).

The constituents of nanoemulsion are biodegradable, such as dietary fiber, which is a kind of polysaccharide and recognized as a functional food in the scientific community (Arnon‐Rips & Poverenov, 2018). Meanwhile, the incorporation and controlled release of tea polyphenols (TPPs) into nanoemulsion can improve the texture, impart food functionality, and extend shelf life (Liang et al., 2017; Onoue et al., 2011). It has been reported that epigallocatechin gallate was the main component of TPPs, which could inhibit the oxidation of food systems by donating hydrogen atoms, trapping free radicals, interrupting chain oxidation reactions, and chelating metal ions (López de Dicastillo et al., 2011). However, the hydrophilicity of TPPs restricts their application and effectiveness in oil‐rich foods. The nanoemulsion can effectively overcome these limitations of antioxidant compatibility, and the presence of nanoparticles increases the tortuosity of the diffusion path to allow the antioxidant to continue to release slowly (Sekhon, 2010).

Fish floss (FF) is a dried aquatic product with a fluffy and light texture similar to that of coarse cotton. It is a nutritious item popularly consumed in Chinese, Indonesian, and Vietnamese dining, especially used as filling for various pastries and buns, and as a snack food on its own. However, FF is susceptible to oxidation and moisture absorption because of the high content of protein and polyunsaturated lipids (Lin et al., 2019). In this study, soluble dietary fiber and TPPs were incorporated into nanoemulsion to fabricate a bioactive edible film on the surface of FF. Its effects on the structural characteristics, water activity, and volatile flavor components have been studied.

## 2 MATERIALS AND METHODS

### 2.1 Materials and reagents

The soluble dietary fiber (SDF) of mung bean (*Vigna radiata* L.) hull was bought from Watson Biotech Co., Ltd. (Hangzhou, China). Canned tuna FF was supplied by Zhejiang Ocean Family Co., Ltd. (Hangzhou, China). The tea polyphenol (TPP), sodium alginate, chitosan, acetic acid, Span 80, Tween 80, gelatin glycerin, and calcium chloride were food‐grade and purchased from Boyan Food Co., Ltd. (Yangzhou, China). Gallic acid, sodium carbonate, magnesium chloride hexahydrate, and potassium chloride were purchased from Sinopharm Chemical Reagent Co., Ltd. (Shanghai, China).

### 2.2 Preparation of nanoemulsion

#### 2.2.1 Emulsification

The coated nanoemulsion (CNE) was prepared according to the formula previously reported by Li et al. (2013) and Liu et al. (2017) with slight modification. Briefly, monocaprylate was mixed with Span 80 at a ratio of 7:3 (w/w) using as oil phase. The W/O microemulsion (W/O‐ME) was obtained by adding TPP (1 ml, 25 mg/ml) to the oil phase (9 ml) dropwise and magnetically stirring at 900 rpm (revolutions per minute) for 20 min at room temperature. Thereafter, a solution (60 ml) containing 0.07% sodium alginate, 3% Tween 80, and 0.04% SDF was prepared and added to the W/O‐ME dropwise. The multiple emulsion (Multi‐E) was formed after ultrasonic homogenization (60 Hz) for 10 min. Finally, 1 ml of CaCl_2_ (0.05%) and 4 ml of chitosan (0.06%) were added to the Multi‐E dropwise in sequence. The CNE was obtained with continuous stirring at 600 rpm for 30 min.

#### 2.2.2 Determination of the entrapment ratio

Two milliliters of CNE was accurately measured and transferred into a volumetric flask (10 ml). A portion of methanol (6 ml) was added to break the uncoated nanoemulsion (UCNE), and ultrapure water was added to the volume. Afterward, the mixture was centrifuged at 3000 *g* for 10 min, and the content of TPP in the supernatant was determined. The encapsulation ratio of TPP was calculated as follows:

$$\text{ER}\;\left(\%\right)=1‐\frac{w_{\text{ut}}}{w_{\text{tt}}}\times 100$$

where ER is the entrapment ratio; *w*_ut_ is the amount of unencapsulated TPP (mg); and *w*_tt_ is the total amount of TPP (mg).

#### 2.2.3 Particle size measurement

The prepared CNE was appropriately diluted with deionized water, and the particle diameter was measured using a Mastersizer 3000E laser particle size analyzer (Malvern, Britain). The pump speed was set at 250 *g*. Each sample was measured in parallel three times, and the results were averaged.

### 2.3 Preparation of NEF solution and NEF‐FF

#### 2.3.1 Preparation of NEF solution

Gelatin solution (5%, w/v) was prepared by the hydration of gelatin in distilled water for 1 h, and glycerin (20%, w/w) was added as a plasticizer to reduce the brittleness of the film. The NEF solution was formulated by dissolving the gelatin solution (25 ml) into the freshly prepared CNE (75 ml) with continuous stirring for 1 h. As a comparison, the NEF solution was obtained by mixing the gelatin solution (25 ml) with TPP aqueous solution (75 ml) to a final concentration of 0.25 mg/ml. Another portion of the gelatin solution (25 ml) was added to ultrapure water (75 ml) and stirred for 1 h to obtain a blank film. To prepare the solid film, the solutions (40 ml) were pipetted to Petri dishes with hot‐air drying (40°C) for 16 h. The films were conditioned in a desiccator at 25°C and 60% relative humidity for 2 days before physiochemical analysis.

#### 2.3.2 Preparation of NEF‐FF

A mass of 10 g FF was accurately weighed and spread on a plate. The film solution was evenly sprayed on the surface of FF. Afterward, the NEF‐FF was placed in an incubator to form a solid film at 50°C. The ratio of film solution to FF was optimized ranging from 0 g/g to 0.25 g/g on the basis of sensory score and water activity.

### 2.4 Sensory evaluation

The sensory evaluation was carried out by a double‐blind method in triplicate. The well‐trained panel consisting of 10 professionals graded the FF samples in terms of color, texture, flavor, and taste, as the evaluation criteria shown in Table S1 (Kazemi et al.. 2020). The product assessment interval required the assessor to rinse the sample with water to avoid interaction effects. The assessment personnel could not communicate and were assigned separately.

### 2.5 Physicochemical analysis

The methods of microscopic analysis, X‐ray diffraction (XRD), water vapor permeability, and water activity were described in Supplementary Materials.

### 2.6 Releasing ability of NEF

Fish floss (FF) is a kind of oily food, and 95% ethanol was recommended as a mimic of such food because of its similar hydrophobicity (Iñiguez‐Franco et al., 2012). An appropriate amount of NEF (10 mg TPP equivalent) was mixed with 30 ml of the simulant in a 50 ml glass bottle. The releasing test was carried out at 25°C in darkness by determining the content of TPP in the simulant at intervals of an hour. All the measurements were performed in triplicate.

The behavior of NEF in the simulated gastrointestinal environment was also tested. The simulated gastric and intestinal juices were prepared according to Minekus's protocol (Minekus et al., 2014). An appropriate amount of NEF (10 mg TPP eq.) was added to 60 ml of simulated juice, and the mixture was stirred at 100 rpm. At hourly intervals, the suspension was centrifuged, and the content of TPP released in the supernatant was determined by a Folin–Ciocalteu method (Slinkard & Singleton, 1977). Briefly, the supernatant (2 ml) was thoroughly mixed with 0.1 ml of Folin–Ciocalteu reagent (10% v/v). Then, 8 ml of sodium carbonate (7.5%, w/v) was added to the mixture. After standing at room temperature for 60 min, the absorbance was measured at 765 nm, and the content of TPP was calculated and expressed as gallic acid equivalent (GAE) (mg)/sample (g).

### 2.7 Analysis of volatile compounds

The volatile compounds of NFF and NEF‐FF were analyzed on a Thermo Trace DSQ II automatic headspace–solid‐phase microextraction (HS–SPME) coupled with gas chromatography–mass spectrometry (GC/MS, Thermo Scientific, MA, USA). The sample (5.0 g) was placed in a 20 ml glass vial and incubated at an extraction temperature of 70°C for 20 min. The volatile compounds were extracted for 30 min and desorbed for 6 min using a divinylbenzene/carboxen/polydimethylsiloxane (DVB/CAR/PDMS) fiber, followed by their separation on a TR‐35MS GC column (30 m × 0.25 mm × 0.25 μm). The experimental conditions were listed as follows: inlet temperature 250°C, carrier gas flow rate 0.8 ml/min, ion source energy 70 eV, transmission line temperature 250°C, ion source temperature 250°C, quadrupole temperature 150°C, and scanning range *m/z* 35–450.

### 2.8 Statistical analyses

Results were expressed as mean ± standard deviation. Differences in means were determined by Duncan's multiple range test and *p* < .05 was deemed statistically significant, using the SPSS 21.0 computer program (SPSS Statistical Software Inc., Chicago, IL, USA). OriginPro 2021 (Originlab, Northampton, MA, USA) was used for graphing. All measurements were performed in triplicate.

## 3 RESULTS AND DISCUSSION

### 3.1 Optimization of CNE

The preparation of CNE relied on the electrostatic adsorption of charged polysaccharide macromolecules such as SDF, chitosan, and sodium alginate and the crosslinking effect of calcium ions. These conditions influenced the stability and slow release ability of the CNE to achieve long‐term antioxidant and prolonged storage periods. The following mainly aimed to optimize the CNE formulation from the aspects of SDF, chitosan, sodium alginate, and calcium ion.

**FIGURE 1:**
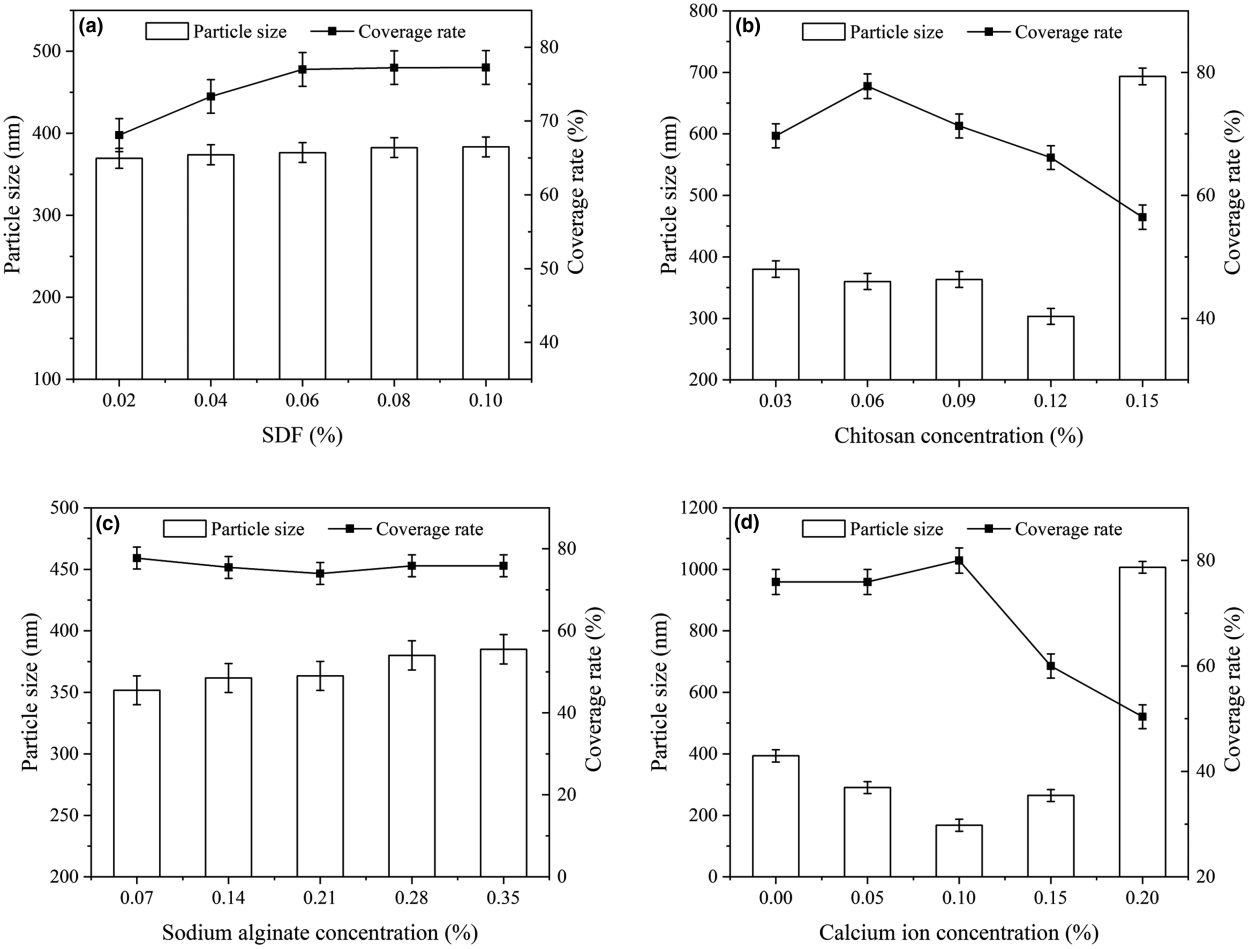
Effect of (a) soluble dietary fiber (SDF), (b) chitosan, (c) sodium alginate, and (d) Ca^2+^ concentration on the entrapment efficiency and particle size of the coated nanoemulsion

The SDF contains charged polysaccharides, such as pectin, which play an important regulatory role in the automatic assembly and coating of nanoemulsion. The concentration of SDF was optimized ranging from 0.02% to 0.10%. As shown in Figure 1a, the coating rate of nanoemulsion reached its highest top (76.99%) at 0.06%, and became constant thereafter. Meanwhile, the particle size of the CNE enlarged in a slow and steady manner along with the increasing addition of SDF. The oversized particle dimension was not conducive to the stability of CNE. Therefore, 0.06% SDF was selected as the optimal concentration.

Chitosan is a kind of deacetylated chitin, which is composed of α‐(1 → 4) glycosidic linking _D_‐glucosamine (deacetyl unit) with *N*‐acetyl‐_D_‐glucosamine (acetylation unit). This positively charged linear polysaccharide exhibits good biocompatibility and excellent film‐forming property (Sarmento et al., 2007). It was observed from Figure 1b that the coating rate increased along with the rise of concentration from 0.03% to 0.06%, and then dropped gradually when the addition of chitosan enhanced from 0.06% to 0.15%. On the contrary, the particle size of CNE reduced gradually at first and then increased sharply. The electrostatic interaction between chitosan and sodium alginate was enhanced when the concentration of chitosan increased in a small scale, resulting in small particle size and high coating rate. However, excessive chitosan would compete with nanoemulsion to a certain extent in the coating. The sodium alginate would partially coat the chitosan instead of the nanoemulsion, resulting in a decreased coating rate. When the concentration of chitosan increased to 0.15%, the ingredients of sodium alginate, chitosan, SDF, and CaCl_2_ would crosslink together and produce flocculent gel precipitate, leading to a sharply increased particle size of CNE and decreased coating rate. Therefore, the concentration of chitosan was set at 0.06%.

Sodium alginate is a negatively charged linear polysaccharide polymer consisting of 1,4 glycosidic linked β‐_D_‐mannuronic acid and α‐_L_‐guluronic acid, which can interact with chitosan, SDF, etc. to achieve coating. The CNE was optimized by varying the concentration of sodium alginate (0.07%, 0.14%, 0.21%, 0.28%, 0.35%). As shown in Figure 1c, the concentration of sodium alginate had little effect on the coating rate of CNE in the tested concentration levels, which indicated that there was enough charge of sodium alginate to crosslink to chitosan and other agents. The particle size increased gradually along with the increase of sodium alginate concentration. A concentration of 0.07% sodium alginate solution was enough to fabricate the edible film.

### 3.2 Crosslinker CaCl_2_

The effect of crosslinker CaCl_2_ on the coating rate and particle size of CNE was studied in the concentration levels from 0% to 0.20%. As shown in Figure 1d, compared with the CNE without calcium ion crosslinker, the addition of a small number of calcium ions had little effect on the coating rate, but it significantly reduced the particle size of CNE, which was probably attributed to the curing effect of calcium ion crosslinker. The smallest particle size and the largest coating rate of CNE were obtained when the concentration of CaCl_2_ reached 0.1%. When the concentration of CaCl_2_ was further increased, the CNE particle grew up to micron level, and the coating rate began to decrease sharply at the same time. This phenomenon could be explained that the excessive calcium ion crosslinker would closely crosslink to the polysaccharide of SDF and sodium alginate, which seriously affected the particle size and coating rate of CNE, resulting in flocculent gel precipitate. Finally, the concentration of crosslinker CaCl_2_ was set at 0.1%. As a consequence, the preferred CNE formulation was 0.06% SDF, 0.06% chitosan, 0.07% sodium alginate, and 0.1% calcium chloride.

### 3.3 Ratio of NEF solution to FF

**FIGURE 2:**
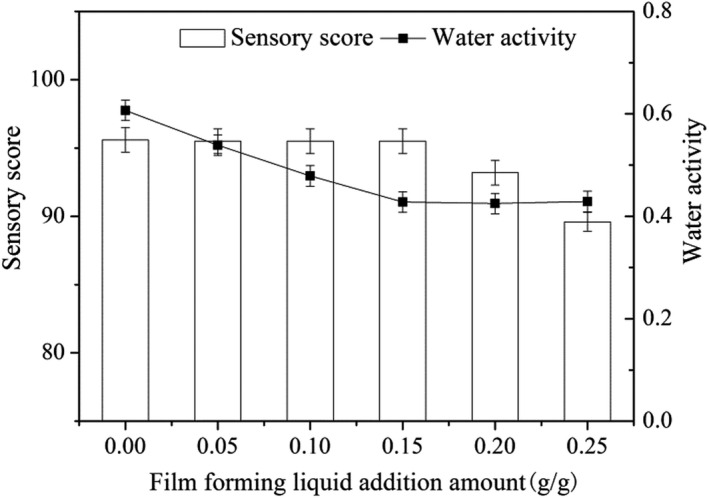
Selection of the amount of film‐forming solution added

The ratio of NEF solution to FF is a crucial factor for the quality of NEF‐FF. The optimized NEF can improve the sensory experience of FF, reduce the water activity, and control the release of antioxidants to extend the shelf life of FF. The quantity of NEF solution used for the preparation of NEF‐FF was optimized. Figure 2 shows the effect of NEF quantity on the sensory experience and water activity of NEF‐FF. It was indicated that the flavor of NEF‐FF could be maintained if the addition of NEF solution was kept below 0.15 g/g. Along with the dosage of NEF further increased, the morphology of FF was held, and the water activity of NEF‐FF was kept at a lower level which was favorable for storage. However, excessive NEF would weaken the taste and mouthfeel. To obtain a high sensory score and appropriate low water activity, a 0.15 g/g NEF solution addition amount was selected to prepare NEF‐FF.

### 3.4 Characterization of NEF

**FIGURE 3:**
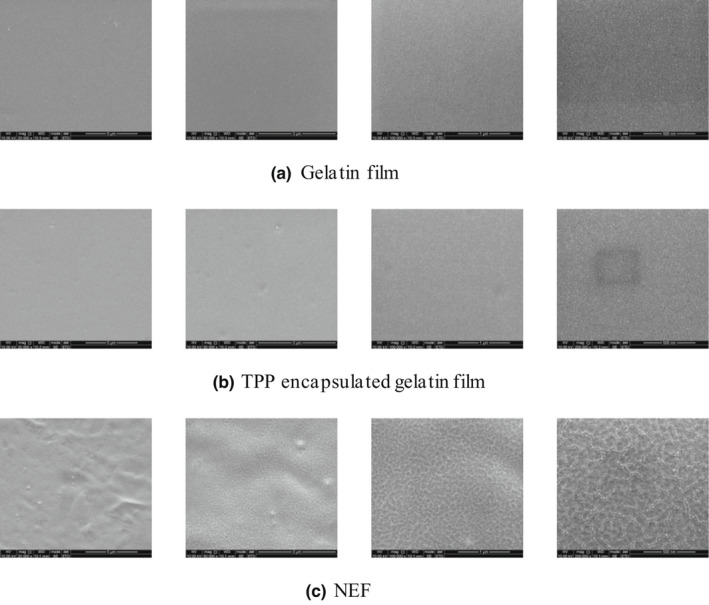
Structure of the edible film surface

The surface structural characteristics of blank gelatin film, TPP‐encapsulated gelatin film, and NEF were measured by SEM, as shown in Figure 3. It was directly observed that there was almost no difference between the blank gelatin film (Figure 3a) and the TPP‐encapsulated gelatin film (Figure 3b). The smooth surfaces of both films indicated that the addition of TPP had no effect on the surface structural characteristic of edible film. It was reported that the TPP was hydrogen‐bonded with gelatin, but this reaction could not be directly observed by SEM. The surface of the NEF (Figure 3c) was relatively rough. Acevedo‐Fani et al. and Shojaee‐Aliabadi et al. also found that the nanoemulsion with oil phase made the microstructure of the edible film coarser than that of the oil‐free edible film, which was attributed to the upward migration of oil droplets during the evaporation process, resulting in rounded hump or protuberance (Acevedo‐Fani et al., 2015; Shojaee‐Aliabadi et al., 2014).

**FIGURE 4:**
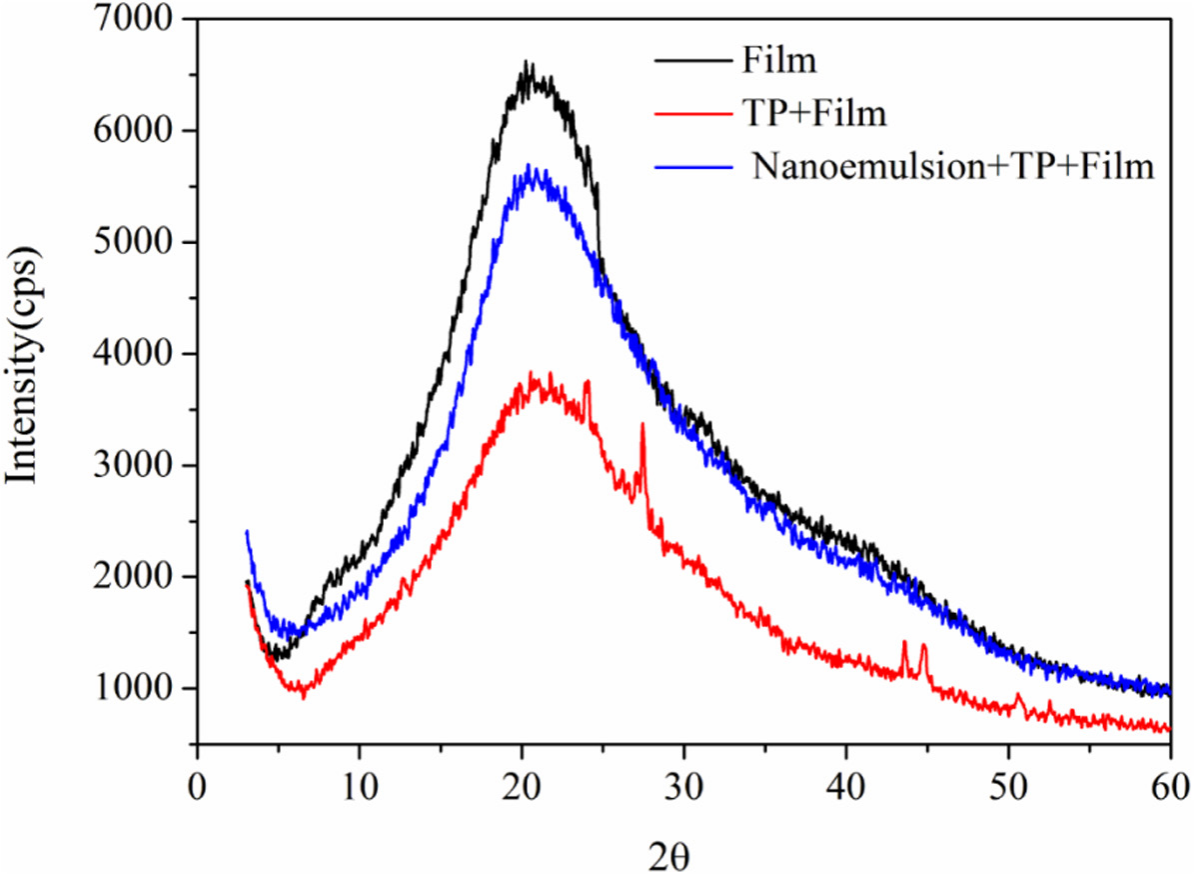
Edible film X‐ray diffraction (XRD) pattern

Differences of crystallinity between the blank gelatin film, TPP‐encapsulated gelatin film, and NEF are depicted in Figure 4. In general, the crystallinity was positively related to the height and sharpness of the peak in the XRD spectrum. The blank gelatin film was regarded as the control group and possessed the highest crystallinity due to the triple helix structure of gelatin. The fortification of TPP decreased the crystallinity of gelatin film significantly, leading to a flat and broad peak in the bottom of the spectrum. It was indicated that the hydrogen bonding between gelatin and TPP reduced the strength of triple helix structure and thus weakened the crystallinity of the film (Wu et al., 2013). The crystallinity of NEF was lower than that of the blank gelatin film, but higher than that of the TPP‐encapsulated gelatin film. It was indicated that the incorporation of nanoparticles destroyed the hydrogen bonds between gelatin molecules, resulting in a decrease in the content of triple helix unit. Arrieta et al. (2014) also reported that the increase of crystallinity and crystallization region indicated a slow release of catechin from the poly(lactic acid) –poly(hydroxybutyrate) blends.

Water vapor permeability (WVP) is an index for measuring the barrier functions of the edible film, and this value can represent the ability of the edible film to regulate the water activity of the food (Babapour et al., 2021). The edible film used in food coatings or packaging materials is applied to prevent or reduce the dehydration of food or to reduce the influence of external moisture. Only a low WVP value can effectively control the transportation of water molecules between the environment and food (Ma et al., 2008).

The blank gelatin film and NEF were placed in a high humidity environment. It was observed from Figure S1 that the WVP of the blank gelatin film rose rapidly up to 2.33% with the prolongation of time, while the WVP of NEF went up in a gradual manner and reached only 0.99% after 5 h. It was indicated that the NEF regulated moisture better than the blank gelatin film. This phenomenon was related to the ratio of hydrophilic and lipophilic matters in films. The blank gelatin film mainly consisted of gelatin, which possessed strong hydrophilicity due to the presence of a large number of hydrophilic hydroxyl groups and amino groups, resulting in a high WVP value of the blank gelatin film. The oil phase presented in film increased the tortuosity for water molecules passing through the film, and thus enhanced the resistance of film to water vapor. The coated nanoemulsion structure of NEF made a more uniform distribution of oil phase, resulting in a better moisture regulation ability of NEF.

The release of antioxidants from NEF is a complex process related to the macromolecular polymer matrix, antioxidant properties, and food characteristics (Mousavian et al., 2020). It has been concluded by XRD analysis that the controlled release ability of NEF was better than that of the TPP‐encapsulated gelatin film. In this section, the controlled release ability of NEF was further investigated under different simulated conditions, including the surface of oily food and the gastrointestinal environment. The results are shown in Figure S2. On the surface of oily food, the TPP was steadily released at a constant rate, and such slow release effect guaranteed a long‐term protection of oily food from oxidation (Chen et al., 2012). In the simulated gastrointestinal environment, the release rates of TPP were much higher. In detail, the release rate of TPP in stomach was 17.80% in the first 1 h and even higher in intestine, which reached 60.12% after 5 h of digestion. It was attributed to the effects of various factors, such as enzyme, pH, etc. In the simulated gastrointestinal fluid environment, the release of TPP contributed to the decomposition of the outer shell of NEF, resulting in speeding up the release rate. The results indicated the superiority of NEF in the controlled release of TPP.

### 3.5 Characterization of NEF‐FF

**FIGURE 5:**
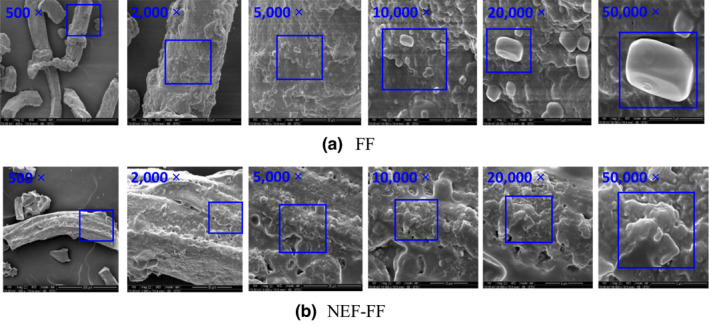
The microstructures of fish floss (FF) (a) and nanoemulsion‐based edible film‐fish floss (NEF‐FF) (b) with the magnification of 500×, 2000×, 5000×, 10,000×, 20,000×, 50,000×, respectively

The surface structure of FF and NEF‐FF was characterized by SEM, as shown in Figure 5. By using a low power lens, both the FF and NEF‐FF displayed fibrous structures. As the magnification increased, there were granular particles that appeared on the surface of the uncoated FF, which might be small crystals of salts or sucrose. In contrast, no granular particles were observed on the surface of NEF‐FF. The NEF solution completely dissolved or coated these small crystals of salts and sucrose, indicating that the NEF had a positive effect on improving the morphology and reducing the rough mouthfeel of FF.

The water activity of FF is the ratio of the partial vapor pressure of water in the atmosphere in equilibrium with FF to that of the atmosphere in equilibrium with water under identical conditions. As shown in Figure S3, in a high humidity environment, the water activity of FF increased rapidly from an initial AW (water activity) value of 0.59 to 0.91 in a short time and then grew up continuously at a constant rate. The initial AW values of TF‐FF and NEF‐FF were significantly lower than that of FF benefiting from the protection of edible film. After different storage periods, the AW of TF‐FF rose significantly, while the NEF‐FF maintained at a low AW value. The growth of AW during the period for each FF was calculated to be in the order of FF (0.32) > TF‐FF (0.26) > NEF‐FF (0.07). The ability to control water activity was tentatively attributed to the presence of oil phase in NEF, which induced the tortuosity of water permeability and enhanced the resistance of NEF to water vapor. Therefore, the treatment of NEF coating effectively prevented the invasion of water molecule to FF.

The effect of NEF on the volatile compounds of FF was studied by HS–SPME–GC/MS. A total of 33 volatile compounds were detected in FF, including hydrocarbons (19), aldehydes (3), ketone (1), alcohols (2), phenol (1), furan (1), and esters (6). The differences in the relative content of each volatile compound between FF and NEF‐FF samples were compared, as shown in Table 1. After film coating, the NEF‐FF displayed a different profile of volatile compounds. Specifically, some compounds, such as 3‐decene, isopropyltoluene, terpinolene, naphthalene, isobornyl acetate, etc., were not detected, because the FF was coated and these compounds could not permeate through the film. Besides, there were also some volatile compounds newly produced, such as 4‐methyl‐tetradecane, 2,5‐dimethyl‐tridecane, 2‐nonanone, 1,3,5‐benzenetriol, 3‐methylphenol, 4‐2,5‐dimethylpyrazine, etc., which were probably due to Maillard reaction and lipid degradation during the NEF coating process.

**TABLE 1:** Analysis of volatile flavor compounds of fish floss (FF) and nanoemulsion‐based edible film‐fish floss (NEF‐FF)

Classification	Time (min)	Flavor substance	Relative percentage (%)	
			FF	NEF‐FF
Hydrocarbons	1.89	Hexane	1.28 ± 0.04^a^	0.37 ± 0.05^b^
	4.21	6‐methyl‐cyclotrisiloxane	1.06 ± 0.07^a^	0.19 ± 0.01^b^
	7.43	Ethylbenzene	2.51 ± 0.09^a^	0.26 ± 0.03^b^
	7.65	1,2‐xylene	2.28 ± 0.11^a^	0.38 ± 0.07^b^
	8.24	3‐decene	0.69 ± 0.06^a^	—
	9.45	Decane	0.83 ± 0.06^a^	0.37 ± 0.05^b^
	12.26	Cyclohexene	15.25 ± 0.39^a^	0.69 ± 0.07^b^
	12.36	10‐methyl‐cyclopentasiloxane	0.39 ± 0.07^a^	0.15 ± 0.01^b^
	12.87	Isopropyltoluene	0.87 ± 0.13^a^	—
	14.74	Terpinolene	4.78 ± 0.31^a^	—
	16.17	Isopropenyltoluene	0.73 ± 0.08^a^	—
	18.40	12‐methyl‐cyclohexasiloxane	0.99 ± 0.17^a^	0.18 ± 0.02^b^
	21.90	Naphthalene	0.44 ± 0.04^a^	—
	22.72	10‐methyl‐nonadecane	—	0.26 ± 0.02^a^
	24.40	5‐methyl‐tetradecane	—	0.57 ± 0.02^a^
	24.56	4‐methyl‐tetradecane	—	2.16 ± 0.11^a^
	24.98	3‐methyl‐tetradecane	—	0.59 ± 0.05^a^
	25.52	2,5‐dimethyl‐tridecane	—	1.96 ± 0.12^a^
	27.12	2,6,10‐trimethyl‐tetradecane	—	—
	25.56	2‐methylnaphthalene	1.11 ± 0.21^a^	—
	26.52	Cypressene	1.13 ± 0.07^a^	—
	27.03	Pentadecane	1.25 ± 0.06^a^	0.59 ± 0.01^b^
	29.62	Hexadecane	1.03 ± 0.21^a^	0.61 ± 0.09^b^
	31.33	Tetradecane	0.42 ± 0.05^a^	0.36 ± 0.07^a^
	33.10	Cypress	0.91 ± 0.32^a^	—
	Total		37.95 ± 0.57^a^	9.69 ± 1.03^b^
Aldehyde	6.09	Hexanal	5.39 ± 0.56^a^	3.69 ± 0.29^b^
	11.70	2‐heptenal	6.61 ± 0.46^a^	5.67 ± 0.10^b^
	16.44	Nonanal	4.35 ± 0.16^a^	3.59 ± 0.18^b^
	Total		16.35 ± 0.99^a^	12.95 ± 0.61^b^
Ketones	8.92	2‐heptanone	2.91 ± 0.11^a^	0.79 ± 0.09^b^
	15.04	2‐nonanone	—	0.69 ± 0.02^a^
	18.97	2‐undecone	—	1.28 ± 0.13^a^
	Total		2.91 ± 0.11^a^	2.76 ± 0.08^a^
Alcohol	11.19	1‐octen‐3‐ol	1.29 ± 0.13^a^	0.32 ± 0.07^b^
	15.96	N‐tridecyl alcohol	—	0.59 ± 0.01^a^
	16.48	2‐hexen‐1‐ol	1.06 ± 0.06^a^	—
	Total		2.35 ± 0.27^a^	0.91 ± 0.10^b^
Phenols	30.30	2,4‐di‐tert‐butyl‐phenol	0.61 ± 0.09^b^	0.98 ± 0.12^a^
	23.50	1,3,5‐benzenetriol	—	0.27 ± 0.06^a^
	24.75	3‐methylphenol	—	0.36 ± 0.03^a^
	Total		0.61 ± 0.09^b^	1.61 ± 0.30^a^
Pyrazine and furan	10.44	2,5‐dimethylpyrazine	—	0.68 ± 0.07^a^
	16.23	2,3‐diethylpyrazine	—	0.56 ± 0.09^a^
	19.59	5‐pentylfuranone	1.71 ± 0.09^a^	0.60 ± 0.05^b^
	Total		1.71 ± 0.09^a^	1.84 ± 0.12^a^
Ester	2.66	Ethyl acetate	2.76 ± 0.11^a^	0.96 ± 0.21^b^
	5.71	Ethyl butyrate	0.34 ± 0.05^a^	0.28 ± 0.03^a^
	11.80	Isobornyl acetate	0.46 ± 0.02^a^	—
	35.72	Ethyl myristate	1.40 ± 0.21^a^	0.68 ± 0.07^b^
	39.19	Salicylate	0.47 ± 0.03^a^	0.11 ± 0.01^b^
	39.74	Ethyl palmitate	0.49 ± 0.04^a^	0.43 ± 0.05^a^
	Total		5.92 ± 0.20^a^	2.46 ± 0.09^b^
Other heterocyclic compounds	31.16	Dodecenyl succinic anhydride	—	1.34 ± 0.14^a^
	Total		0	1.34 ± 0.14^a^

Note

Different letters within the same row indicate significant differences (*p* < .05).

Although the hydrocarbons were the most abundant volatile compounds in both FF and NEF‐FF, they tended to have high sensory thresholds, and thus exerted a limited effect on food flavor (Curran et al., 2005). Similarly, the thresholds of alcohols and esters were also high, and their contribution to flavor was neglectable. Aldehydes were produced by the oxidative degradation of triglycerides and polyunsaturated fatty acids, most of which presented grassy and fishy odor. The NEF could continuously release TPPs which inhibited and/or slowed down the oxidation process, and thus maintained the aromatic flavor of FF. The NEF treatment increased the diversities of ketones, phenols, and pyrazine furans. The ketones and pyrazine furans might be resulting from the Maillard reaction of sugars in FF and the degradation and oxidation of lipids. The latter volatile compounds had low threshold values and produced nutty, meaty, and chocolate‐like flavors, which contributed significantly to the flavor of NEF‐FF (Chang, Lin, et al., 2021). The increase of phenolic flavor was attributed to the release of polyphenols from NEF. Conclusively, the unfavorable factors of external moisture and microorganism activity were blocked. The NEF increased the contents of ketones, phenols, and pyrazines, which had a positive regulating effect on the overall flavor of FF.

## 4 CONCLUSIONS

To extend the shell life of FF during storage, a CNE‐based edible film was fabricated on the surface of FF using an optimized formula developed in this study. The specific film structure promised an improved morphology of FF and reduced crystallinity of the film. Meanwhile, the NEF effectively improved the flavor and taste of FF, controlled the release of TPP, and reduced the water activity during the storage. As a conclusion, the CNE‐based NEF solution made a film with good physicochemical performance and could be further employed to make films on the packaging of FF to enhance the quality. Nevertheless, further studies are needed to explore the mechanism involved in the formation and function of the film.

## CONFLICT OF INTEREST

The authors declare that there is no conflict of interest.

## ETHICAL APPROVAL

The sensory analysis test that contained human participants was approved by the Ethics Committee of Zhejiang Gongshang University. We have obtained written informed consent from all study participants. All of the procedures were performed in accordance with the relevant policies in China.

## Supporting information

Supplementary Material

## Data Availability

The data that support the findings of this study are available from the corresponding author upon reasonable request.
