# Supplementary material for: Fabrication and characterization of tea polyphenol W/O microemulsion‐based bioactive edible film for sustained release in fish floss preservation
Source: Food Sci Nutr. 2022 Apr 21;10(7):2370–80. doi: 10.1002/fsn3.2845 (PMC9281946; doi:10.1002/fsn3.2845)
Supplement: Supplementary file 1 — Supplementary Material [file FSN3-10-2370-s001.doc]

**Supplementary materials**

**Fabrication and characterization of bioactive edible film for the preservation of fish floss**

**Contents**

**Physicochemical analysis**

**Microscopic observation**

**X-ray diffraction**

**Water vapor permeability**

**Water activity**

**References**

**Tables**

1. The sensory scoring standards.

**Figures**

1. Water vapor transmission rate of edible film.
2. In vitro simulated release of tea polyphenols coated with nanoemulsion.
3. Control of water activity of fish floss by the edible film.

**Physicochemical analysis**

**Microscopic observation**

The morphology of NEF was characterized by scanning electron microscopy (SEM, model S4800, Hitachi, Japan). Briefly, the film piece was fixed on a copper holder with double-sided tape and coated with a layer of gold (5 min, 2 mbar). Subsequently, the surface and microstructure of NFF and NEF-FF were directly observed under an acceleration voltage of 15 kV and magnifications of 100×, 1000×, and 5000×.

**X-ray diffraction**

X-ray diffraction (XRD) analysis was performed using an X-ray diffractometer ((D/max-TTRIII, Rigaku; Tokyo, Japan) with a background free sample holder. The loading voltage was 40 kV, the current was 100 mA, and the scanning speed was 4^◦^/min. In continuous mode, the data was collected over an angular range from 5^◦^ to 50^◦^ with step size of 0.02^◦^ and step time of 5 s.

**Water vapor permeability**

The water vapor permeability test was carried out using method reported by Mei et al. (2020) with some modifications. The sample (EF or NEF) was sealed with paraffin in a methyl methacrylate cell containing 4 g of anhydrous calcium chloride (0% relative humidity). The cell was then placed at in an incubator with a humidity of 90% ± 2% 25 ^◦^C, and the quality was measured periodically. The WVP was calculated as follows:

$$WVP(\%)=\frac{w-w_{0}}{w_{0}}\times100\%$$

Where: *WVP* is water vapor permeability; *w* is the weight of the film sample after water vapor permeation (g); w0 is the weight of the original film sample (g).

**Water activity**

The fish floss sample was stored in an incubator with 80% humidity at 25 ^◦^C. The water activity was tested during the storage period from day-0 to day-4 using an HD-5 intelligent water activity meter (Furbs, China), which was calibrated with saturated magnesium chloride. A certain amount of sample (5 g) was accurately weighed and placed in a sample dish that was connecting to the instrument. The water activity was measured by a sensor inside, and the value could be directly read from an automatically printed report.

**References**

Mei, L. X., Nafchi, A. M., Ghasemipour, F., Easa, A. M., Jafarzadeh, S., & Al-Hassan, A. A. (2020). Characterization of pH sensitive sago starch films enriched with anthocyanin-rich torch ginger extract. *International Journal of Biological Macromolecules, 164*, 4603-4612. https://doi.org/10.1016/j.ijbiomac.2020.09.082

Table S1. Sensory scoring standards

| Item | Comprehensive weight | Standard | Score |
| --- | --- | --- | --- |
| Color | 25% | Very light or very dark | 0-60 |
|  |  | Lighter or darker | 61-80 |
|  |  | Golden color | 61-80 |
| Organizational morphology | 25% | broken tissue, difficult to form | 0-60 |
|  |  | Surface damage | 61-80 |
|  |  | Surface integrity | 81-100 |
| Flavor | 25% | Insufficient taste, not obvious | 0-60 |
|  |  | Has a special flavor of fish floss, the taste is normal | 61-80 |
|  |  | Flavored, good taste | 81-100 |
| Taste | 25% | Harder or softer | 0-60 |
|  |  | The taste is normal, slightly worse | 61-80 |
|  |  | Good taste and crispy feeling | 81-100 |

| 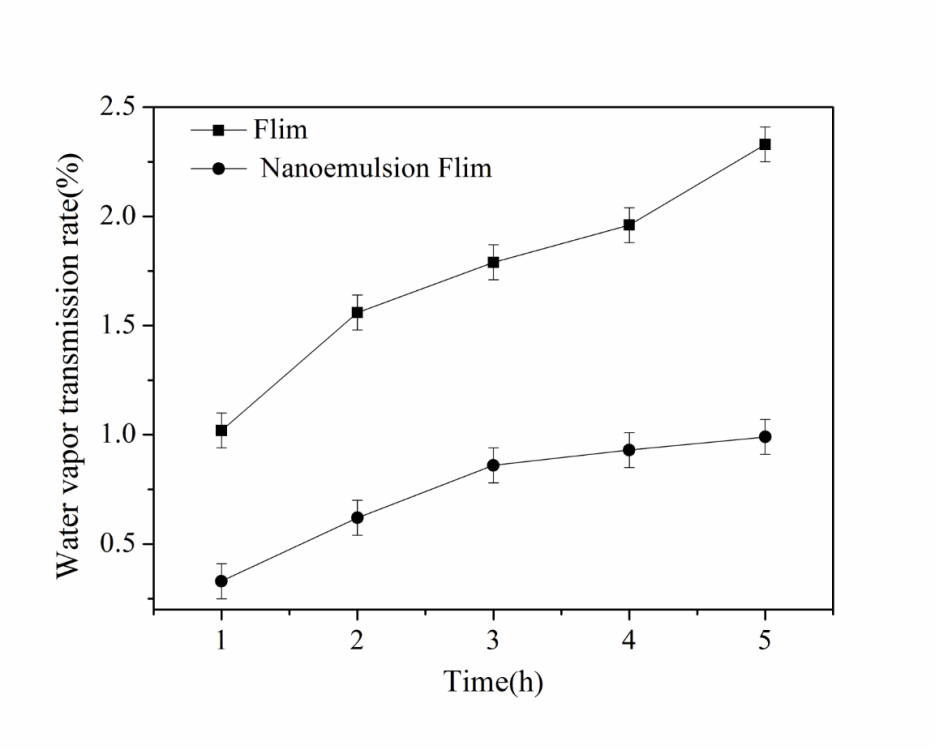 |
| --- |
| Figure S1 |

| 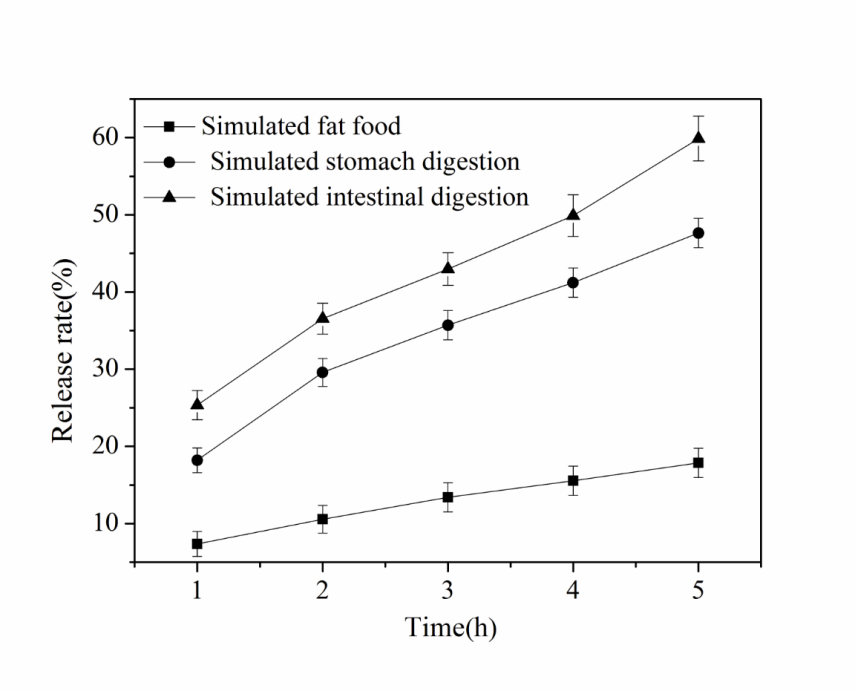 |
| --- |
| Figure S2 |

| 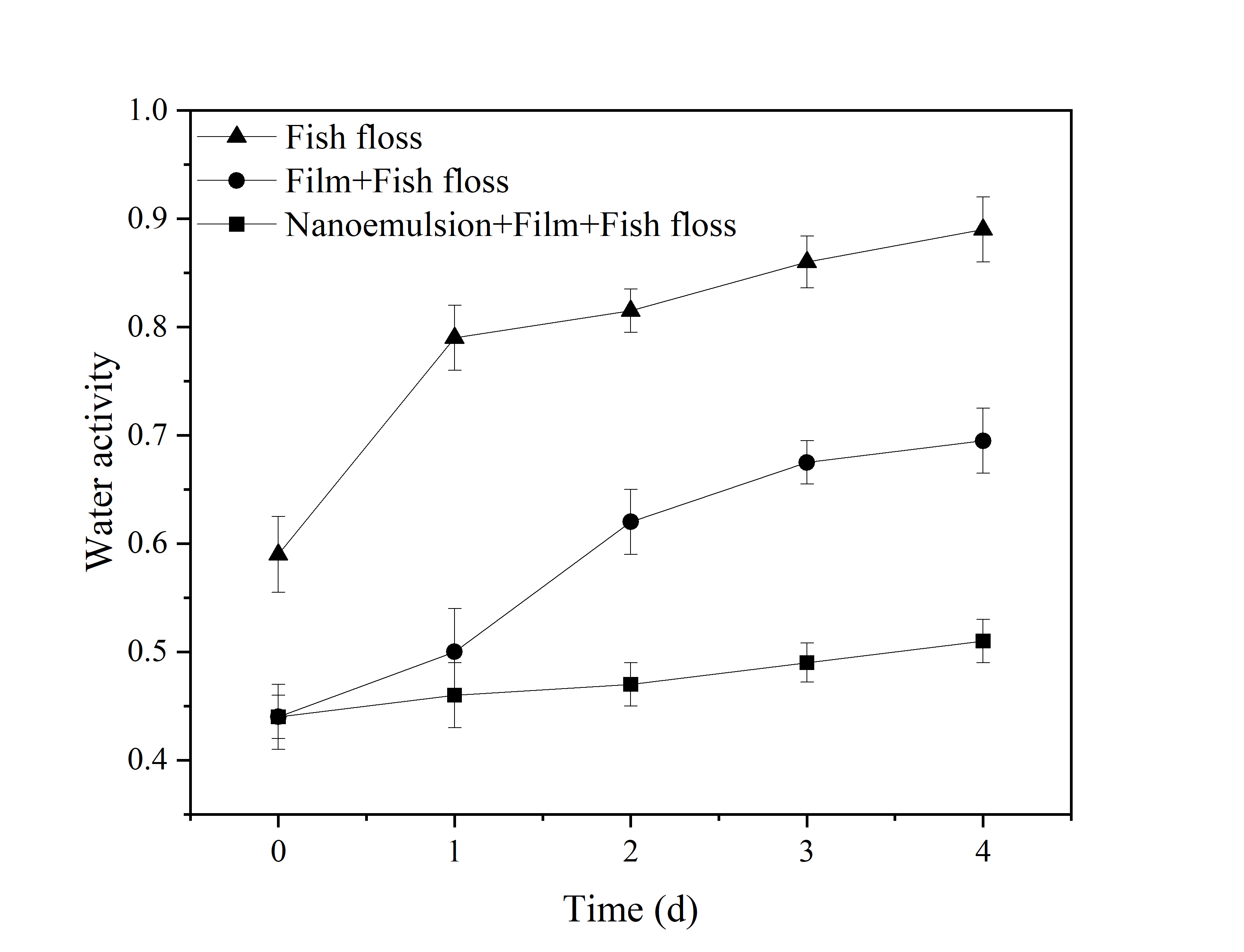 |
| --- |
| Figure S3 |
